# Survival of Bacterial Strains on Wood (*Quercus petraea*) Compared to Polycarbonate, Aluminum and Stainless Steel

**DOI:** 10.3390/antibiotics9110804

**Published:** 2020-11-13

**Authors:** Ju-Chi Chen, Muhammad Tanveer Munir, Florence Aviat, Didier Lepelletier, Patrice Le Pape, Laurence Dubreil, Mark Irle, Michel Federighi, Christophe Belloncle, Matthieu Eveillard, Hélène Pailhoriès

**Affiliations:** 1 Laboratoire Innovation Matériau Bois Habitat Apprentissage (LIMBHA), Ecole Supérieure du Bois, 7 rue Christian Pauc, 44000 Nantes, France; r01643012@ntu.edu.tw (J.-C.C.); tanveer.munir@esb-campus.fr (M.T.M.); mark.irle@esb-campus.fr (M.I.); christophe.belloncle@esb-campus.fr (C.B.); 2 Your ResearcH-Bio-Scientific, 307 la Gauterie, 44430 Le Landreau, France; florenceaviat@gmail.com; 3 Laboratoire MiHAR EE 1701 S, Institut de Recherche en Santé 2, Université de Nantes, 22 boulevard Benoni-Goullin, 44200 Nantes, France; lepelletier.didier@gmail.com; 4 EA 1155 IICiMed, Institut de Recherche en Santé 2, Université de Nantes, 22 boulevard Benoni-Goullin, 44200 Nantes, France; patrice.lepape@chu-nantes.fr; 5 PAnTher, INRAE, École Nationale Vétérinaire, Agro-alimentaire et de l’alimentation Nantes Atlantique (Oniris), Université Bretagne Loire (UBL), 44307 Nantes, France; laurence.dubreil@oniris-nantes.fr; 6 UMR INRA 1014 SECALIM, Oniris, route de Gachet, CS 40706, CEDEX 03, 44307 Nantes, France; michel.federighi@oniris-nantes.fr; 7 CRCINA, Inserm, Université de Nantes, Université d’Angers, Angers, 44200 Nantes, France; MaEveillard@chu-angers.fr; 8 Laboratoire de Bactériologie-Hygiène, Centre Hospitalier Universitaire, 4 rue Larrey, 49933 Angers, France; 9 Laboratoire HIFIH, UPRES EA3859, SFR 4208, Université d’Angers, 49933 Angers, France

**Keywords:** *Quercus petraea*, sessile oak, wood, antimicrobial materials, healthcare-associated pathogens, hospital environment

## Abstract

Healthcare-associated infections (HAI) remain a burden in healthcare facilities, environmental surfaces being a potential reservoir for healthcare-associated pathogens. In this context, exploration of materials with potential antimicrobial activities represents a way forward for the future. Here, we explored the survival of four bacterial species commonly involved in HAI (*Acinetobacter baumannii*, *Enterococcus faecalis*, *Klebsiella pneumoniae*, *Staphylococcus aureus*), on oak versus three other materials (aluminum, polycarbonate, stainless steel). Twenty microliters of each bacterial suspension (approximatively 10^7^ bacteria) were deposited on each material. Bacterial counts were measured by grinding and culturing on day 0, 1, 2, 6, 7 and 15. Analyses were performed in triplicate for each material and each time evaluated. It appeared that the bacteria viable count decreased rapidly on transversal and tangential oak compared with the other materials for all bacterial species. Furthermore, no difference was noticed between transversal and tangential oak. These results underline the potential for use of oak materials in healthcare facilities, a consideration that should be supported by further investigations.

## 1. Introduction

Healthcare-associated infections (HAI) remain a continuously increasing cause of morbidity and mortality [1]. European point prevalence surveys, from 2016 to 2017, realized on 310,755 patients in acute care hospitals (ACH) and 117,138 residents in long-term care facilities (LTCF) in 28 countries, estimated that 6.5% of patients in ACH and 3.9% of residents in LTCF had at least one HAI [2]. The contamination of hospital surfaces plays an important role in the transmission of healthcare-associated pathogens, which contaminate work surfaces and medical equipment in the rooms of patients. Indeed, some bacterial pathogens, such as Methicillin-Resistant *Staphylococcus aureus* (MRSA), Vancomycin-Resistant *Enterococcus* sp., carbapenemase-producing Enterobacteriaceae or *Acinetobacter baumannii*, possess the ability to survive from days to weeks in the hospital environment [3,4,5]. Furthermore, microbial contamination can occur quickly after disinfection, or even persist despite cleaning [3].

In this context, materials with potential antimicrobial activity appear as a major research axis for controlling HAI. For years, wood has been neglected in healthcare buildings due to its porous nature, the problem of whether wood surfaces can be considered as hygienic or not remaining a strong issue. At the same time, this organic material features the advantages of being a renewable resource. The use of wood material for the indoor construction of healthcare buildings has been shown to have a positive influence on the mental health and wellbeing of patients and medical staff, but also on physiological parameters [6,7]. Recently, some growing evidence attests that some wood species such as oak, have antimicrobial properties [8,9,10,11]. Indeed, in a preceding study, *Quercus petraea* has been shown to have antimicrobial activity, using agar diffusion method, against a panel of MRSA and Methicillin-Susceptible *Staphylococcus aureus* (MSSA) isolates [10]. Another study of Munir et al. has pointed out that the antimicrobial activity of *Quercus petraea*, tested with wood disc diffusion method, was stronger on *S. aureus* isolates than on *A. baumannii* ones [9]. This antimicrobial activity was also greater using discs cut respecting transverse section, than tangential or radial sections, giving rise to the suspicion of a better delivery of the antimicrobial compounds using the transversal cut [9]. The underlying mechanisms of this antimicrobial activity are not completely understood and are assumed to be a combination of the anatomical parameters of this material and its chemical composition. Furthermore, if many studies have explored the antimicrobial properties of the extractive content of oak wood, few data concerning the bacteria survival on wood materials are available. The aim of this study is to investigate the survival of different bacterial species (*K. pneumoniae*, *A. baumannii*, *S. aureus* and *E. faecalis*) on oak, cut respecting transversal and longitudinal sections, compared to other materials (polycarbonate, aluminum and stainless steel).

## 2. Material and Methods

### 2.1. Material Preparation

Four materials were tested in this experiment: sessile oak (*Quercus petraea*), polycarbonate (Makrolon^®^, Bayer, Auvergne-Rhone-Alpes, France), stainless steel 316 TI and aluminum (alloy). Wood discs were manufactured from samples of *Quercus petraea*, coming from mature trees grown in France. The wood samples were obtained from heartwood and were dried at 21 °C and 60% relative humidity during four weeks [12]. The discs were prepared from the wood pieces using a laser cutting machine while respecting transversal and tangential sections. Wood discs of 2–4 mm thickness and 9–10 mm diameter were obtained. Discs of stainless steel (squares 10 × 10 mm and 1 mm of thickness), aluminum (squares 10 × 10 mm and 2–3 mm of thickness) and polycarbonate (discs 9–10 mm diameter and 2–3 mm of thickness) were manufactured and sterilized (134 °C for 20 min for steel, aluminum and stainless steel 316Ti; and 121 °C for 20 min for polycarbonate) before the experiments.

### 2.2. Bacterial Strains

Four bacterial strains were used in this study: *Klebsiella pneumoniae* American Type Culture Collections (ATCC) 700603 producing an extended-spectrum beta-lactamase (ESBL) (SHV-18), *Staphylococcus aureus* KSKS7326 (methicillin-resistant *S. aureus*), *Acinetobacter baumannii* ATCC 19606, and *Enterococcus faecalis* ATCC 51299 harboring the *vanB* gene of resistance to glycopeptides. The bacterial strains were grown on Columbia sheep blood agar plates (Bio-Rad, Marnes-La-Coquette, France) 24 h at 35 ± 2 °C.

### 2.3. Survival Assay

The four microorganisms were cultured overnight 12–18 h in brain heart infusion (BHI) broth (bioMérieux, Marcy l’Etoile, France). A 3 mL aliquot of the overnight broth culture was centrifuged 5 min at 12,000× *g* (miniSpin plus, Eppendorf, Hamburg, Germany). The supernatant was removed, the bacterial cells were then washed with 3 mL of distilled water, centrifuged and resuspended in 3 mL of distilled water. A volume of 20 μL (approximatively 10^7^ Colony Forming Units (CFU)) of this suspension was then homogeneously deposited on the surfaces of transversal oak (Oak T), of tangential oak (Oak L), of steel, of aluminum, of stainless steel and of polycarbonate. Each bacterial suspension was inoculated on 21 specimens of each material, which were placed in covered petri dishes. The chips were maintained in a room with temperature from 18 to 24 °C, and 29 to 64% of relative humidity (RH). The determination of the viable counts was performed on the initial bacterial suspension, and then on the specimens on day 0, day 1, day 2, day 3, day 6, day 7 and day 15. For each time and on each material, the determination of the viable bacteria counts was performed in triplicate.

### 2.4. Determination of the Viable Bacteria Counts

The viable bacteria counts were determined on the initial bacterial suspensions before the inoculation on the chips. These counts were determined by a spread plate method with 10-fold serial dilutions of the initial bacterial suspensions on Columbia sheep blood agar plates (Bio-Rad, Marnes-la-Coquette, France).

The viable bacteria counts were also determined on the specimens on day 0 (after 1.5 to 3 h of drying), day 1, day 2, day 3, day 6, day 7 and day 15. Three specimens were used for each count. Each specimen was washed with 4 mL of distilled water, and mixed for 30 s with 5 stainless steel beads in a mixer mill (MM 400, Retsch, Haan, Germany) at a vibration frequency of 30/s. Then, appropriate volume of the rinsate was plated on sheep blood agar plates (direct plating of 10 μL, 100 μL or 1 mL of the solution or 10-fold serial dilutions following the expected CFU count). The viable counts were determined by counting the CFU after an incubation of 24 to 48 h at 35 ± 2 °C, following the aspect of the colonies. Each time of sampling, materials were tested in triplicate and the mean of the three viable bacteria counts was calculated.

### 2.5. Statistical Analysis

Statistical analysis was performed using Kruskal Wallis test with Dunn’s Multiple Comparison Test using GraphPad Prism (version 5.02 for Windows, GraphPad Software, San Diego, CA, USA). For statistical analysis, the viable counts that were ≤4 CFU were estimated as 4 CFU.

## 3. Results

**Figure 1:**
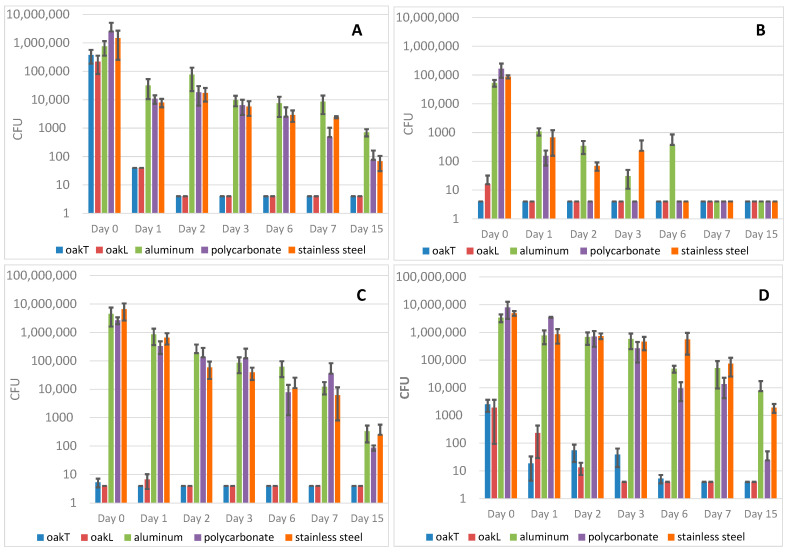
Average Colony Forming Units (CFU) of *Klebsiella pneumoniae* ATCC 700603 (**A**), *Acinetobacter baumannii* ATCC 19606 (**B**), *Enterococcus faecalis* ATCC 51299 (**C**) and *Staphylococcus aureus* KSKS7326 (**D**) on five materials. Blue bars represent transversal oak, orange bars represent tangential oak, grey bars represent aluminum, yellow bar represent polycarbonate and green bars represent stainless steel. The black lines represent the standard deviation (SD). *y* axis: bacterial CFU. *x* axis: day of determination of the viable counts.

The results of bacteria count for each time, each material, and each bacterial strain are presented on Table 1 and Figure 1.

**Figure 2:**
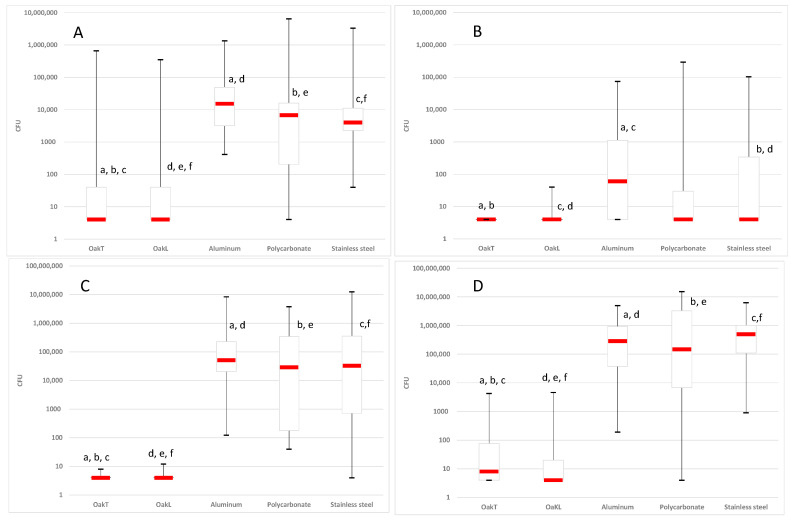
Box plot representing viable bacteria count distribution (from day 0 to day 15) for *Klebsiella pneumoniae* ATCC 700603 (**A**) with significant differences reported according to Dunn’s Multiple Comparison Test: (a) oakT vs. aluminum *p* ≤ 0.001; (b) oakT vs. polycarbonate *p* ≤ 0.01; (c) oakT vs. stainless steel *p* ≤ 0.001; (d) oakL vs. aluminum *p* ≤ 0.001; (e) oakL vs. polycarbonate *p* ≤ 0.01; (f) oakL vs. stainless steel *p* ≤ 0.001, *Acinetobacter baumannii* ATCC 19606 (**B**) with significant differences reported according to Dunn’s Multiple Comparison Test: (a) oakT vs. aluminum *p* ≤ 0.001; (b) oakT vs. stainless steel *p* ≤ 0.01; (c) oakL vs. aluminum *p* ≤ 0.001; (d) oakL vs. stainless steel *p* ≤ 0.05 (**C**) with significant differences reported according to Dunn’s Multiple Comparison Test: (a) oakT vs. aluminum *p* ≤ 0.001; (b) oakT vs. polycarbonate *p* ≤ 0.001; (c) oakT vs. stainless steel *p* ≤ 0.001; (d) oakL vs. aluminum *p* ≤ 0.001; (e) oakL vs. polycarbonate *p* ≤ 0.001; (f) oakL vs. stainless steel *p* ≤ 0.001, and *Staphylococcus aureus* KSKS7326 (**D**) with significant differences reported according to Dunn’s Multiple Comparison Test: (a) oakT vs aluminum *p* ≤ 0.001; (b) oakT vs. polycarbonate *p* ≤ 0.001; (c) oakT vs. stainless steel *p* ≤ 0.001; (d) oakL vs. aluminum *p* ≤ 0.001; (e) oakL vs. polycarbonate *p* ≤ 0.001; (f) oakL vs. stainless steel *p* ≤ 0.001 on five materials. *y* axis: bacterial CFU.

Overall, the distribution of viable bacteria counts from day 0 to day 15 was significantly different between wood (transversal and tangential oak) and the other materials for nearly every bacterial strain studied. For *K. pneumoniae*, this distribution was significantly different between the wood material and aluminum (*p* < 0.0001), polycarbonate (*p* < 0.01) and stainless steel (*p* < 0.001). Concerning *S. aureus*, the significance degrees were *p* < 0.0001, *p* < 0.0001, and *p* < 0.01 respectively. All differences between wood and other materials had a significance degree of *p* < 0.0001 for *E. faecalis*. For *A. baumannii*, the significance degrees were *p* < 0.001 between oak and aluminum materials, *p* < 0.01 between transversal oak and stainless steel, and *p* < 0.05 between tangential oak and stainless steel (Figure 2).

As the bacterial counts were often very different between wood and other materials at day 0, we compared the decrease of bacterial counts between the different materials from the counts at day 0. For *K. pneumoniae*, a 4-log decrease occurred at day 1 on transversal and tangential oak. This 4-log decrease was recorded at day 7 on polycarbonate and stainless steel for this bacterium, whereas it was not recorded at day 15 on aluminum. For *S. aureus*, a 2-log decrease was observed at day 2 on both wooden materials. The same decrease was recorded at day 6 for polycarbonate and at day 7 for stainless steel. Concerning *A. baumannii* and *E. faecalis*, the comparison of the bacterial count decrease was very difficult because bacterial counts were very low for wood material at day 0, comparing with aluminum, polycarbonate and stainless steel.

## 4. Discussion and Conclusions

Environmental surfaces contamination provides a reservoir for the persistence of pathogens and can be a source for HAI. This study investigated the survival of different bacterial species (*K. pneumoniae*, *A. baumannii*, *S. aureus* and *E. faecalis*) on oak wood and other commonly used indoor hospital surfaces (stainless steel, aluminum, polycarbonate).

The results showed that all the bacterial strains tested here survived less time on wood surfaces compared to the other materials. This observation can be explained by the antimicrobial activity of wooden material, that can be associated both with the anatomical structure of this material and its chemical composition [13]. The antimicrobial properties of oak wood species against *S. aureus*, *K. pneumoniae*, *A. baumannii* and *E. faecalis* have been documented in the literature [8,9,10,11,12,14,15]. In these studies, only the antimicrobial properties of the extractive content of oak wood have been documented. However, the antimicrobial properties of wood are a combination of chemical, but also of anatomical parameters which create unsuitable growth conditions for many pathogenic bacteria. This assertion is supported by the fact that in previous studies, *A. baumannii* and *E. faecalis* showed mild susceptibility to the diffusion-based antimicrobial properties of solid oak wood [9,12]. However, in the current investigation, the two *E. faecalis* and *A. baumannii* isolates survived less than 24 h on transversal and tangential oak. This supports the observation that antimicrobial properties of wood are not only a result of the extractives activity but also of the physical properties of this live material. In the same way, although *Escherichia coli* has been reported as resistant to agar-diffused antibacterial oak compounds [12], Milling et al. reported that bacterial titer of *E. coli* decreased faster on oak wood sawdust compared with poplar, maple, spruce, beech and plastic [16]. These results attest the strong antimicrobial properties of oak wood material, which is also supported by the rapid decrease of the viable bacteria count on wood material for all the species tested.

In this study, the total viable cell count was significantly lower in oak (transversal and tangential) than for polycarbonate, stainless steel and aluminum, with a faster decrease of CFU bacterial counts on wood materials. However, this decrease is more difficult to compare as the bacterial counts were very different on day 0 between wood material and the other ones. The only exception was the absence of difference in survival of *A. baumannii* between oak and polycarbonate. Indeed, for *E. faecalis*, *K. pneumoniae* and *S. aureus*, the minimum detectable limit (MDL) was reached within 0 to 2 days on oak materials whereas this MDL was not attained in 15 days on polycarbonate, stainless steel and aluminum. For *A. baumannii* isolate, the MDL was reached within one day on oak species, within 2 days on polycarbonate and within 6 and 7 days on stainless steel and aluminum. The short time to MDL is a substantial advantage for raw wood material, needing further exploration to test the survival of other microorganisms.

In initial results (day 0), a low number of bacteria were observed from both categories of wood samples for *A. baumannii* (≤4 CFU on transversal oak and 16 CFU on tangential oak on day 0), E. faecalis (5 CFU on transversal oak and ≤4 CFU on tangential oak on day 0) and *S. aureus* (2.5 × 10^3^ CFU on transversal oak and 2.9 × 10^3^ CFU on tangential oak on day 0), comparing with the other materials. This lower microbial recovery from wood could have been associated with the porous nature of this material. However, the “grinding” technique used in this article for the recovery of bacteria is supposed to minimize the effect of porosity on the recovered bacteria, comparing with swabbing, planning or brushing techniques and is presented as an optimal recovery method [17]. Furthermore, the absence of difference in bacterial survival between transversal-cut and tangential-cut oak does not support the hypothesis of a strong impact of the porosity, transversal section being more porous than tangential section. In an experiment of Vainio-Kaila et al., the rapid decrease of *E. coli* and *Listeria monocytogenes* on wood compared with glass surfaces has been explored [18]. In this last study, the bacterial counts recovered from scots pine heartwood samples, using a vortex protocol, began to decrease after 2 h, as compared to 24 h on glass samples. In this same article, the possibility for bacteria of tightly adhering to wood materials was studied by incubating the wood samples overnight in BHI broth after the vortex protocol. Only one sample on 15 tested with *L. monocytogenes*, and 0 samples on 15 tested with *E. coli* had positive broth culture after negative result with vortex protocol [18]. This result demonstrates that the porous structure of wood is not an explanation for the rapid decrease of bacterial concentration on wood surfaces. Also, the fact that some bacteria such as *S. aureus*, *A. baumannii* and *E. faecalis* are concerned by this phenomenon and not *K. pneumoniae*, should lead to the exploration of differences of adherence capacity on wood depending on the bacterial species. The rationale for this rapid decrease of CFU on wood material, as a potential result of physical and chemical mechanisms should also be further explored.

In this experiment, one limit is that the ageing process of the different materials has not been taken into account. It should be interesting to conduct an experiment to test the different materials over a long period of time. Indeed, some individual parts of the materials could be placed indoor in healthcare building and tested in real conditions to control the microbial contamination and the material behavior over the months. Furthermore, the same experiment as realized in this study should be carried out in different temperature and humidity conditions to test the antimicrobial capacity of wood over different environmental conditions. Indeed, it has been suggested that low temperature (4–6 °C) and high humidity (>70%) are factors associated with a longer bacterial persistence on inanimate surfaces [4]. Such conditions should be then also tested in the case of oak. At last, the antimicrobial activity of other types of wood should be tested in comparison with oak. Thus, some antimicrobial effect has been reported for pine heartwood surfaces in comparison with glass [18]. So, it would be interesting to test a panel of different wood species to compare the effect of these ones on the bacterial survival.

This study underlines the low survival length of several bacterial species involved in HAI on sessile oak. Some investigations are needed to explore the behavior of other species, and the physical and chemical mechanisms involved in such phenomenon. Our results are also encouraging regarding the possible use of oak material in healthcare facilities, but further experiments should be needed to assess wood material behavior in these conditions. 

## Figures and Tables

**Table 1:** Viable counts of *Klebsiella pneumoniae* American Type Culture Collection (ATCC) 700603, *Staphylococcus aureus* KSKS7326, *Acinetobacter baumannii* ATCC 19606, and *Enterococcus faecalis* ATCC 51299 on the different materials tested.

Bacteria	Material	Initial Suspension (CFU/20 μL)	Day 0 (CFU)	Day 1 (CFU)	Day 2 (CFU)	Day 3 (CFU)	Day 6 (CFU)	Day 7 (CFU)	Day 15 (CFU)
*Klebsiella pneumoniae* ATCC 700603	Oak T	1.6 × 10^7^	3.8 × 10^5^	4.0 × 10^1^	≤4	≤4	≤4	≤4	≤4
	Oak L		2.2 × 10^5^	4.0 × 10^1^	≤4	≤4	≤4	≤4	≤4
	Aluminum		7.5 × 10^5^	3.2 × 10^4^	7.7 × 10^4^	9.8 × 10^3^	7.5 ×10^3^	8.6 × 10^3^	7.1 × 10^2^
	Polycarbonate		2.5 × 10^6^	1.1 × 10^4^	1.8 × 10^4^	6.4 × 10^3^	2.5 × 10^3^	4.8 × 10^2^	7.6 × 10^1^
	Stainless steel		1.5 × 10^6^	8 × 10^3^	1.7 × 10^4^	5.7 × 10^3^	2.9 × 10^3^	2.4 × 10^3^	6.8 × 10^1^
*Staphylococcus aureus* KSKS7326	Oak T	2.4 × 10^7^	2.5 × 10^3^	8	5.4 × 10^1^	3.8 × 10^1^	5	≤4	≤4
	Oak L		2.9 × 10^3^	2.3 × 10^2^	1.3 × 10^1^	≤4	≤4	≤4	≤4
	Aluminum		3.4 × 10^6^	7.7 × 10^5^	6.7 × 10^5^	5.8 × 10^5^	4.8 × 10^4^	5.1 × 10^4^	7.6 × 10^3^
	Polycarbonate		7.9 × 10^6^	3.5 × 10^6^	7.2 × 10^5^	2.7 × 10^5^	9.8 × 10^3^	1.4 × 10^4^	2.4 × 10^1^
	Stainless steel		4.9 × 10^6^	8.5 × 10^5^	7.4 × 10^5^	4.6 × 10^5^	5.6 × 10^5^	7.4 × 10^4^	1.9 × 10^3^
*Acinetobacter baumannii* ATCC 19606	Oak T	1.4 × 10^6^	≤4	≤4	≤4	≤4	≤4	≤4	≤4
	Oak L		1.6 × 10^1^	≤4	≤4	≤4	≤4	≤4	≤4
	Aluminum		5.3 × 10^4^	1.1 × 10^3^	3.4 × 10^2^	3.0 × 10^1^	3.7 × 10^2^	≤4	≤4
	Polycarbonate		1.6 × 10^5^	1.5 × 10^2^	≤4	≤4	≤4	≤4	≤4
	Stainless steel		7.8 × 10^4^	9.1 × 10^2^	6.0 × 10^1^	3.4 × 10^2^	≤4	≤4	≤4
*Enterococcus faecalis ATCC* 51299	Oak T	2.1 × 10^7^	5	≤4	≤4	≤4	≤4	≤4	≤4
	Oak L		≤4	6	≤4	≤4	≤4	≤4	≤4
	Aluminum		4.5 × 10^6^	8.6 × 10^5^	1.8 × 10^5^	8.4 × 10^4^	6.2 × 10^4^	1.2 × 10^4^	3.3 × 10^2^
	Polycarbonate		2.7 × 10^6^	3.3 × 10^5^	1.3 × 10^5^	1.3 × 10^5^	7.8 × 10^3^	3.5 × 10^4^	4.6 × 10^2^
	Stainless steel		6.5 × 10^6^	6.5 × 10^5^	5.9 × 10^4^	3.9 × 10^4^	1.1 × 10^4^	6.2 × 10^3^	2.6 × 10^2^
